# Silicon Mitigates Negative Impacts of Drought and UV-B Radiation in Plants

**DOI:** 10.3390/plants11010091

**Published:** 2021-12-28

**Authors:** Anja Mavrič Čermelj, Aleksandra Golob, Katarina Vogel-Mikuš, Mateja Germ

**Affiliations:** 1 Biotechnical Faculty, University of Ljubljana, Jamnikarjeva ulica 101, 1000 Ljubljana, Slovenia; aleksandra.golob@bf.uni-lj.si (A.G.); katarina.vogelmikus@bf.uni-lj.si (K.V.-M.); mateja.germ@bf.uni-lj.si (M.G.); 2 Jozef Stefan Institut, Jamova 39, 1000 Ljubljana, Slovenia

**Keywords:** abiotic stress, crop plants, antioxidants, Si fertilization, oxidative stress

## Abstract

Due to climate change, plants are being more adversely affected by heatwaves, floods, droughts, and increased temperatures and UV radiation. This review focuses on enhanced UV-B radiation and drought, and mitigation of their adverse effects through silicon addition. Studies on UV-B stress and addition of silicon or silicon nanoparticles have been reported for crop plants including rice, wheat, and soybean. These have shown that addition of silicon to plants under UV-B radiation stress increases the contents of chlorophyll, soluble sugars, anthocyanins, flavonoids, and UV-absorbing and antioxidant compounds. Silicon also affects photosynthesis rate, proline content, metal toxicity, and lipid peroxidation. Drought is a stress factor that affects normal plant growth and development. It has been frequently reported that silicon can reduce stress caused by different abiotic factors, including drought. For example, under drought stress, silicon increases ascorbate peroxidase activity, total soluble sugars content, relative water content, and photosynthetic rate. Silicon also decreases peroxidase, catalase, and superoxide dismutase activities, and malondialdehyde content. The effects of silicon on drought and concurrently UV-B stressed plants has not yet been studied in detail, but initial studies show some stress mitigation by silicon.

## 1. Introduction

Plants are exposed to various biotic and abiotic stress factors. How abiotic factors such as drought and UV-B radiation impact plants are currently under more intense investigation due to the increasing threat of drought due to climate change. Water shortages result in lower yields of crop plants and limited crop growth, which, in turn, reduces food production. Ozone depletion due to atmospheric pollution also means that higher levels of UV-B radiation are reaching the Earth’s surface. UV radiation is an essential factor for plant growth, but in excess, it can lead to oxidative damage to cells [1,2].

Silicon (Si) treatment of plants has been shown to mitigate some of the negative impacts of drought and enhanced UV-B radiation. In plants exposed to UV-B, Si can increase the activity of the photosynthetic apparatus, decrease the transpiration rate, increase the antioxidant capacity [3,4], and lower the concentrations of reactive oxygen species (ROS) [5] and protective phenol substances, in comparison to plants exposed to UV-B without Si treatment [4]. Similarly, during drought, Si can increase plant growth, relative water content, photosynthesis rate, and chlorophyll content, and affects other physiological responses [6]. This review presents current knowledge and studies about the effect of Si fertilization on mitigation of stress in plants caused by drought and/or excessed UV-B radiation, which often occur together. Though there are many drought and Si related review articles [7,8,9], the UV-B stress and Si and especially the joint effect of drought and UV-B stress and Si fertilization has not been studied in detail yet.

## 2. Silicon

As indicated, Si is an element that can alleviate biotic and abiotic stress in plants. Si can occur in various forms, and it is particularly abundant in the Earth’s crust, as it represents 28.8% of the continental crust [10]. Si is found in the different fractions of soils, including the solid and liquid phases, and it can be absorbed onto soil particles. The liquid phase of the Si dissolved in soil contains monosilicic and polysilicic acids and complexes with inorganic, organic, and organosilicon compounds [11]. Although Si is ubiquitous, plants can take up Si only in the form of monosilicic acid. Therefore, fertilization with Si is recommended, especially for croplands. Large amounts of plant-available Si can be removed from the soil by Si-accumulating plants, such as sugarcane, rice, and wheat. The removal of these plants from the fields lowers amount of silicon in soil [11,12].

Two different types of Si transporter [Low Silicon 1 (Lsi1) and 2 (Lsi2)] involved in the uptake and distribution of Si have been identified. Lsi1 is a Si permeable channel belonging to the Nod26-likemajor intrinsic Protein III subgroup of the aquaporin membrane protein family, with a distinct selectivity. On the other hand, Lsi2 is an efflux Si transporter belonging to an uncharacterized anion transporter family. Lsi1 and Lsi2 are localized to the plasma membrane but show different expression patterns depending on the plant species [13]. Different tissue or cellular localizations of these transporters are associated with different levels of Si accumulation [13]. Besides Lsi1 and Lsi2, Lsi6 is an influx transporter which transports Si from xylem to the shoot cells [14].

Silicon is considered an essential element for the diatom algae (*Bacillariophyceae*) and for horsetail (*Equisetaceae*), which is a vascular plant [15]. Other plant species are Si accumulators, or intermediate types, or Si excluders, with these plant classes defined according to the Si:Ca ratio in their tissues [16]. The plants that have higher Si concentrations are pteridophytes, bryophytes, and monocotyledons, with the monocotyledons including in particular plants from the families *Cyperaceae* and *Poaceae.* Generally, dicotyledons do not accumulate Si, although some belong to the intermediate class (e.g., cucumber, pumpkin) [16].

**Figure 1:**
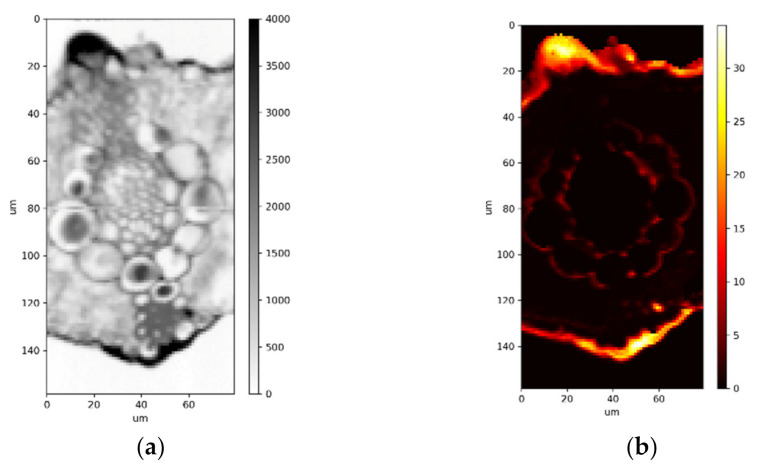

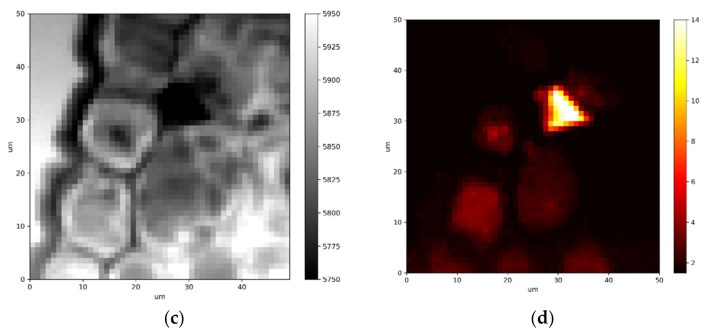
Localization of Si in leaves (**a**) an X-ray absorption image of common reed (*Phragmites australis*) leaf cross-section recorded at TwinMic beamline of synchrotron Elettra depicting X-ray dense regions (black) corresponding to biomineralized leaf areas. (**b**) An X-ray fluorescence image of Si distribution (colour bar in wt%) of common reed (*Phragmites australis*) leaf cross-section recorded at TwinMic beamline of synchrotron Elettra depicting Si accumulation in leaf epidermal region. Data are taken from [19]. (**c**) An X-ray absorption image of tea plant (*Camellia sinensis*) leaf cross-section recorded at TwinMic beamline of synchrotron Elettra depicting X-ray dense regions (black) corresponding to biomineralized leaf areas. (**d**) An Si-rich phytolith in leaf mesophyll of a tea plant (colour bar in wt%). The epidermis in incrusted with Ca (not shown).

In grasses and sedges Si is manly localized on the leaf surface in prickle hairs, epidermis and cuticle [17]. It can be also found in a form of phytoliths in various tissues, especially in dicotyledonous plants. Two types of Si deposition, epidermal and in a form of phytoliths in leaves are shown in Figure 1. Si is known to affect the uptake and translocation of mineral nutrients in different plant species [18].

Plants also use Si to strengthen their structural support and to defend against biological (e.g., herbivory, pathogens) and abiotic (e.g., drought, UV radiation, salinity, metal toxicity) stresses [20,21].

## 3. Drought

With the present environmental changes that are underway, the environmental conditions are becoming less predictable, and in the future, temperatures and CO_2_ concentrations are likely to rise worldwide. There will be more areas with drought, which will cause stress to the plants. In addition, biotic stress and high salinity will become more frequent [22]. Drought and water shortage are stress factors that affect the normal growth and development of plants. These can have an immense negative impact on ecosystems and agricultural regions that provide food for humans and animals [23]. Long periods of drought can reduce wetlands, and result in more extensive fires, faster spread of diseases and pests, and loss of biodiversity. Drought can reduce water quality and diminish crop growth and yield. As this can lead to famine, it is essential to minimize the effects of drought on crop plants by understanding the plant processes during water deficiency [23,24].

### 3.1. Plant Damage by Drought

The primary indicator of drought stress is the production of ROS, for example singlet oxygen (^1^O_2_), superoxide (O_2_^∙−^), hydrogen peroxide (H_2_O_2_), and hydroxyl radicals (OH^∙^). Those substances damage cells, which can lead to cell, and ultimately plant, death [25].

The photosynthesis rate of plants is also affected by drought [26]. Closing stomata to reduce water loss results in lower gas exchange in the leaf, and consequently lower CO_2_ assimilation. With reduced mesophyll capacity, Rubisco is inactivated [27], photorespiration is increased, and the chloroplast electron transport chain (which reduces NADP+ into NADPH), is over-reduced [28]. This is seen as a decrease in photochemical efficiency of PSII [29]. Severe drought stress also results in reduction in the photosynthetic rate, maximal photochemical efficiency of PSII, and leaf pigment contents [26]. Drought also leads to reduction in plant growth through effects on cell division and cell enlargement and differentiation, and complex interactions between these processes [30]. The contents of N, P and K have also been shown to be lowered by drought stress in sugar beet [31].

### 3.2. Plant Protection from Drought

Many plant species, such as succulents, are adapted and tolerant for long periods of drought due to their thick tissue and waxy cuticle. However, drought-susceptible species can be found in deserts and arid environments, as other plants have developed mechanisms that help them to cope with water shortages [32].

The first response of plants to lower water availability is avoidance. Plants can actively avoid water loss by closing the stomata and reducing stomatal conductance [33], combined with leaf curling. Plants can also increase production of the wax layer on the leaves [34]. Wax prevents evaporation of water from leaf tissues and reduces water loss.

If severe drought conditions occur, the level of drought stress is higher, which means that the plant needs to tolerate lower water availability. Tolerance is possible with osmotic adjustment, by increasing the levels of different sugars or proline [35]. In addition, an increased root-to-shoot ratio represents a drought strategy, because this way, the plant enhances the root system [36]. Larger surface area of roots and connections with mycorrhizal fungi enables the plant to increase its water intake and nutrient absorption [37]. The possible responses of plants to drought also include cell-wall modifications [38,39], reorganization of metabolism [40], and activation of their antioxidant system. Drought stress increases the levels of the antioxidants ascorbate and glutathione [25]. These antioxidants can scavenge excessively produced toxic ROS and reactive nitrogen species [41,42].

### 3.3. Drought and Silicon

Drought and its relations to Si have been well studied, although there are still many unexplained plant processes and responses. In this review, we have collected mainly the recently published reports on this topic, which are overviewed in Table 1. It has been frequently reported that Si can reduce stress caused by different abiotic factors, including drought [21]. Stress reduction is possible through different mechanisms. Physically, Si that is taken up by the plant can deposit in the form of SiO_2_ on the leaf apoplast, and consequently reduce the evapotranspiration rate and osmotic stress [43].

Si stimulates the antioxidant defense systems to decrease oxidative stress. Modification of osmolytes and phytohormones is seen by the change in the levels of proline, abscisic acid, jasmonic acid, salicylic acid, auxins (e.g., indole-3-acetic acid (IAA)), and gibberellic acid, among others [43]. It was found that Si increases proline content in sugarcane (*Saccharum officinarum* L.) during drought stress and increases ascorbate acid peroxidase activity after 30 days of stress. This suggests that Si increases ascorbate acid peroxidase activity to diminish ROS production [44]. On the other hand—peroxidase, catalase, and superoxide dismutase activities were decreased in mango plants (*Mangifera indica* L.) with Si supplementation under drought stress compared to stressed plants without Si supplementation [6]. This outcome could indicate that Si mediates drought-induced production of ROS, which is in line with the study on tomatoes (*Solanum lycopersicum* L.). In this research ROS accumulation increased under drought stress, but with Si treatment, ROS accumulation does not occur because of the Si-mediated energy dissipation [45]. Si treatment reduced malondialdehyde content in water-stressed wheat (*Triticum aestivum* L.), which indicated decreased lipid peroxidation [46]. Application of Si to the plants under water stress increases the content of total soluble sugars, proline, ascorbic acid, and glutathione in wheat, which might provide antioxidant defense against drought [46]. In some studies on rice (*Oryza sativa* L.), a decrease in proline content was found in Si treated plants exposed to drought [47]. Addition of Si induced different responses of plants regarding to proline accumulation which could be beneficial for drought exposed plants.

Plants under drought stress decrease their concentrations of IAA, cytokinins, and gibberellic acid, and increase their concentration of abscisic acid in mango plants [6]. Si addition promotes increased levels of IAA, gibberellic acid, and cytokinins, and decreases the levels of abscisic acid [6].

On a physiological and biochemical level, Si increases nutrient uptake and modifies gas exchange attributes, which leads to an increase in the photosynthetic rate. Treatment of water-stressed tomato plants and Kentucky bluegrass (*Poa pratensis* L.) with Si positively affects their photosynthesis, compared to those without added Si [48,49]. Si diminishes the reduced levels of maximum photochemical efficiency (*F_v_/F_m_*), effective quantum efficiency, actual photochemical quantum efficiency (*ϕ_PSII_*), photochemical quenching coefficient (*q_P_*), and photosynthetic electron transport rate [48,50]. In addition, the *PetE*, *PetF*, *PsbP*, *PsbQ*, *PsbW*, and *Psb28* genes are related to photosynthesis, and their expression was down-regulated under water deficiency, but up-regulated by Si [48]. Si enhanced the net photosynthetic rates in tomato plants and wheat under drought stress [45,46,50]. Cao et al. [45] suggested that Si improves the light energy distribution between PSI and PSII in plants subjected to drought stress. Exogenous application of Si also increased the tolerance to water deficiency in maize (*Zea mays* L.) by enhanced photosynthetic efficiency, stomatal conductance, and cell membrane integrity [51].

Silicon treatment increases maize and cantaloupe (*Cucumis melo* var. *reticulatus* L. Naud. cv. Galia) tolerance to drought by increased growth and numbers of leaves per plant [51,52]. Fruit length, diameter, flesh thickness, and overall fruit yield were also significantly higher when Si was applied to cantaloupe during drought [52]. For mango, addition of Si increased relative growth rate, net assimilation rate, and relative water content, compared to the plants under water stress and the control plants [6]. Similarly, Si addition to water stressed Kentucky bluegrass, increased the net photosynthesis, leaf water contents and turf quality [49]. Zhang et al. [48] and Alzahrani et al. [46] reported that Si improved plant growth under water stress in tomato and wheat. On the contrary, Thorne et al. [53] indicated that Si does not significantly affect growth during drought stress in wheat.

Silicon also increases the water potential and the relative water content in leaves of sugarcane and wheat during drought stress [44,46]. Additionally, Si increases the content of chlorophylls and carotenoids under water stress, compared with control tomato and mango plants [6,48].

During drought stress, treating plants with Si enhances the plant Si concentrations in high and low Si-accumulating wheat cultivars [53]. If present at high levels in the soil, Si can be accumulated in the above-ground plant tissues even in nonaccumulator species (i.e., oilseed rape [*Brassica napus* L.]), and can improve water uptake under drought stress [54]. Foliar application of Si decreases the accumulation of Ni^2+^, Cd^2+^ and Cr^3+^ in maize leaves and grain if the plants are under drought stress by metal-contaminated irrigation water [51].

Si might also trigger the transcription of genes that are related to antioxidant defense, photosynthesis, osmotic adjustment, lignin, and suberin metabolism, although those connections are yet to be confirmed [55].

**Table 1:** Overview of studies on the relationship between drought and silicon (Si).

Plant Species	Silicon Treatment		Drought Duration	Results (Si Treated vs. Not Si Treated)	Reference
	Source	Concentration			
Rice (*Oryza sativa* L.)	Calcium and magnesium silicate	0, 350 kg Si ha^−1^	-	Decreased proline content	Mauad et al., 2016 [47]
Sugarcane (*Saccharum officinarum* L.)	Calcium magnesium silicate	0, 600 kg Si ha^−1^	30/60 days	Increased ascorbate acid peroxidase (30 days), proline (30 days); decreased malondialdehyde	Bezerra et al., 2019 [44]
Wheat (*Triticum aestivum* L.)	Potassium silicate	0, 2, 4, 6 mM Si	45 days	Increased total soluble sugars, proline, ascorbic acid, glutathione, net photosynthetic rate, plant growth, relative water content; decreased malondialdehyde content	Alzahrani et al., 2018 [46]
	Sodium silicate	1.5 mM Si/0.2, 0.9, 1.8 mM Si	6 weeks (soil)/4 weeks (hydroponics)	Increased Si concentration	Thorne et al., 2021 [53]
Maize (*Zea mays* L.)	Sodium silicate	0, 2, 4 mM Si	Two summer seasons	Increased growth, number of leaves per plant, photosynthesis efficiency, stomatal conductance, cell membrane integrity; decreased accumulation of Ni^2+^, Cd^2+,^ Cr^3+^	Abd El-Mageed et al., 2020 [51]
Kentucky bluegrass (*Poa pratensis* L.)	Sodium silicate	0, 200, 400, and 80 0 mg Si L^−1^	20 days	Decreased the leaf C/N ratio; increased photosynthesis, leaf water content, relative growth rate, leaf green color, root/shoot ratio, and turf quality	Chen et al., 2014 [49]
Tomato *(Solanum lycopersicum* L.)	Potassium silicate	2.5 mM Si	7 days	Increased growth, chlorophyll and carotenoid content, maximum photochemical efficiency, effective quantum efficiency, actual photochemical quantum efficiency, photochemical quenching coefficient, and electron transport rate; up-regulation of genes related to photosynthesis	Zhang et al., 2018 [48]
	Sodium silicate	0.6 mM Si	12 days	Increased net photosynthetic rate; decreased reactive oxygen species	Cao et al., 2020 [45]
		0.6, 1.2, 1.8 mM Si	12 days	Increased maximum photochemical efficiency, electron transport rate, net photosynthetic rate	Cao et al., 2015 [50]
Mango (*Mangifera indica* L.)	Potassium silicate	1.5 mM Si	-	Increased indole-3-acetic acid, gibberellic acid, cytokinin levels, relative growth rate, net assimilation rate, relative water content, chlorophyll and carotenoids contents; decreased abscisic acid levels, and peroxidase, catalase, superoxide dismutase activities	Helaly et al., 2017 [6]
Cantaloupe (*Cucumis melo* var. *reticulatus* L. Naud. cv. Galia)	Silicic acid	0, 100, 200 and 400 kg Si ha^−1^	30 days	Increasing growth and number of leaves per plant, fruit length, diameter, flesh thickness, and overall fruit yield	Alam et al., 2020 [52]
Oilseed rape (*Brassica napus* L.)	Orthosilicic acid tetraethyl ester	3.4 mM Si	10 days	Improved water uptake	Saja-Garbarz et al., 2021 [54]

## 4. Ultraviolet Radiation

The sun is a source of energy for plants. Photosynthetic plants convert this light energy into chemical energy through photosynthesis, for wavelengths between 400 nm and 700 nm. This range thus represents the photosynthetically active radiation [56]. The most efficient visible light for photosynthesis is within the range of the best absorption for chlorophyll a and chlorophyll b. These wavelengths are in the blue (450–500 nm) and red (610–760 nm) spectra [57,58]. Wavelengths from 100 nm to 400 nm are known as ultraviolet (UV) radiation, and are divided into three ranges: UV-A, UV-B, and UV-C. UV-C radiation (100–280 nm) is very harmful, but it is entirely absorbed by the atmosphere. UV-B radiation (280–315 nm) is mainly absorbed in the ozone layer, but the amount of radiation that reaches the Earth’s surface can be a stress factor for plants [59]. UV-A radiation (315–400 nm) is not absorbed in the ozone layer and is also the least dangerous of the UV ranges for organisms, due to its lower energy [58,59].

### 4.1. Plant Damage by UV-B Radiation

UV-B radiation is essential for plant growth and metabolism. However, high UV-B exposure can cause damage to plant cells, which is then reflected in the physiological processes of the whole plant. Generally, dicotyledons are more sensitive to UV-B radiation than monocotyledons [60]. UV-B can induce DNA damage and mutations in DNA replication [61,62]. Staxén et al. [63] reported that UV-B radiation affects the plant cell cytoskeleton by causing breaks in cortical microtubules, and in some cases, this can inhibit cell division. UV-B also degrades amino acids, which leads to inactivation of proteins and enzymes [64]. Damage to lipids in plant cell membranes is also caused by UV-B radiation in the presence of oxygen, which is known as lipid peroxidation [1]. Lipid peroxidation can be defined by measuring the malondialdehyde content, which is formed through oxidation and degradation of polyunsaturated fatty acids [65]. UV-B can destroy pigments in the photosynthetic apparatus, such as chlorophylls and carotenoids, and consequently lower the fluorescence of photosystem (PS)II [66,67,68]. It has been shown that UV-B degrades IAA and can down-regulate genes associated with IAA activity [69,70]. IAA is one of the vital plant hormones of the auxin class, and its shortage results in stem growth reduction [70]. A decrease in the activity of Rubisco (an enzyme essential for carbon fixation) of UV-B–exposed plants leads to lower carbon dioxide fixation and oxygen formation, as was reported by Kataria et al. [60]. UV-B–treated barley, wheat, cotton, sorghum, and amaranth contain less chlorophyll than the untreated plants [60,71]. UV-B radiation can result in smaller specific leaf weights [72]. UV-B can affect the photosynthesis rate also indirectly by reducing stomatal conductance, changing leaf anatomy [73], and increasing leaf thickness [74], which can change the light penetration into the leaf, and thus change the morphology of the canopy [59].

### 4.2. Plant Protection from UV Radiation

Plants have evolutionary developed morphological structures to prevent UV radiation from entering into the shoot tissues and physiological mechanisms to avoid and repair the damage caused by UV. The first morphological barrier against UV radiation is the epidermis, with various structures as cuticle and trichomes. Epidermal cells usually contain UV-absorbing compounds such as cinnamoyl esters, flavones, flavonols, and anthocyanins which synthesis increases in the presence of UV-B radiation to prevent damage in photosynthetically active mesophyll [75,76,77]. The trichomes behave as optical filters, screening out wavelengths that could damage sensitive tissues. Protection from strong visible radiation is also afforded by increased surface light reflectance [78].

When penetrating into the cells UV-B rays generate free radicals and ROS, which cause damage to DNA, proteins, and lipids. Protection against surplus UV radiation can result in elimination of free radicals through enhanced antioxidative enzyme activity that protects the cell and its vital processes and DNA repair mechanisms [79,80].

The enzymatic system that alleviates the negative effects of ROS includes superoxide dismutase, catalase, glutathione reductase, peroxidase, and ascorbate acid peroxidase [79,80]. Superoxide radicals can be transformed into hydrogen peroxide by superoxide dismutase, and later into water by catalase, glutathione peroxidases and peroxiredoxins [81]. The non-enzymatic system includes ascorbic acid, glutathione, carotenoids, proline, polyamines, and phenols [80,82].

### 4.3. UV-B and Silicon

Not many studies on the relationships between UV-B stress and mitigation of stress effects by Si supplementation can be found in the literature. Studies regarding the addition of Si or Si nanoparticles have been reported for crop plants such as rice, wheat, and soybean (*Glycine max* L.) (Table 2). Such studies have shown that Si can reduce the negative effects of UV radiation through increased antioxidant capacity [3,4] and lower concentrations of ROS [5].

Availability, and accessibility, of Si can mitigate the harmful effects of UV-B radiation on plants. It has been shown that addition of Si in the form of potassium silicate (K_2_SiO_3_) mitigates UV-B damage to wheat seedlings (*Triticum aestivum* L.) through an increase in plant antioxidant compounds and Si levels in leaves [3]. Si treatment increased total plant biomass and contents of chlorophylls (a + b), soluble sugars, anthocyanins, and flavonoids, and reduced superoxide radical (O^2−^) production and malondialdehyde content (which indicates lipid peroxidation) in the wheat seedlings [3].

Mihaličová Malčovská et al. [5] exposed maize seedlings to short-term UV-B radiation. The seedlings were grown hydroponically and treated with Si or left untreated, as the control. In control plants, after UV-B exposure, the content of ROS and thiobarbituric acid reactive substances increased, along with a small increase in the content of total phenols and flavonoids. After UV-B exposure, the maize treated with Si showed only a small increase in flavonoid content, which indicated stress mitigation by Si. Schaller et al. [83] reported that an excess of Si decreased phenol content in leaves and increased its content in the culm. This suggests that phenols and Si have the same functionality in UV protection.

In UV-B–exposed wheat, high levels of superoxide radical and H_2_O_2_ have been reported; therefore, lipid peroxidation and electrolyte leakage were increased [84]. Shen et al. [85] reported that treating UV-B stressed soybean with Si resulted in decreased levels of catalase, superoxide dismutase and peroxidase activities. Tripathi et al. [84] also showed differences in the anatomical properties of the leaves. UV-B radiation reduced leaf thickness, size of mesophyll cells, and lignification in the metaxylem vessels. UV-B–exposed wheat showed damage to chloroplasts. Addition of Si and Si nanoparticles diminished the damaging impact of UV-B on the leaves. Moreover, the addition of Si and Si nanoparticles improved lignification and suberization of bundle sheath cells and metaxylem vessels [84].

Shen et al. [86] simulated UV-B radiation at 30% stratospheric ozone depletion on soybean and showed that UV-B–treated plants gain less biomass than the controls. UV-B–treated plants also contained more leaf N and P, and less leaf Mg and Ca. UV-B increased the allocation of P, K, and Ca to the roots. In these soybeans, the addition of Si increased the uptake of P and Mg and favored the allocation of P and Ca to the roots.

**Table 2:** Overview of studies on the relationships between UV-B radiation and silicon (Si).

Plant Species	Silicon Treatment		UV Treatment		Results (Si Treated vs. Not Si Treated)	Reference
	Source	Concentration	Level	Duration		
Wheat (*Triticum aestivum* L.)	Potassium silicate	0, 100, 200, 400 mg SiO_2_/kg substrate	25%/50% ozone depletion	-	Increased antioxidant compound contents, Si concentration in leaves, total biomass, and contents of chlorophyll (a + b), soluble sugars, anthocyanins, flavonoids; reduced superoxide radical (O^2−^) production and malondialdehyde content	Yao et al., 2011 [3]
	Si, Si nanoparticles	10 µM Si/Si nanoparticles	Ambient and enhanced UV	2 days, 6/8 h/day	Increased fresh and dry mass	Tripathi et al., 2017 [84]
Rice (*Oryza sativa* L.)	Silicic acid	1.5 mM Si	UV-B, 10 W	30 h	Increased soluble and insoluble UV-absorbing compounds in epidermis; brown spots in plants without Si	Li et al., 2004 [87]
	Sodium silicate	0/5 mM Si	UV-B, 10.27 W m^−2^	15/30 min	Decreased reactive oxygen species, thiobarbituric acid reactive substances, total phenols and flavonoid content	Mihaličova Malčovska et al., 2014 [5]
Soybean (*Glycine max* L.)	Sodium silicate	1.7 mM Si	30% ozone depletion	1 week, 8 h/day	Increased P and Mg uptake and dry mass (in one cultivar)	Shen et al., 2009 [86]
				1 week	Increase in net photosynthetic rate; decrease in proline content, lipid peroxidation, osmolyte leakage	Shen et al., 2010 [4]
		1.7/2.55 mM Si	15%, 30% ozone depletion	1 week, 8 h/day	Decreased catalase, superoxide dismutase and peroxidase activities	Shen et al., 2010 [85]

## 5. Silicon, UV-B, and Drought

Drought and high UV-B radiation in natural ecosystems often occur simultaneously, so knowing how their joint action affects plants is essential. The combination of UV-B, drought, and Si addition is not well known, with only a few studies carried out to date. Shen et al. [4] investigated the effects of Si on soybean seedlings under drought and UV-B radiation. Both of these stress factors caused membrane damage by lipid peroxidation and osmolyte leakage, which were reduced with Si application. Under UV-B and drought stresses, Si also decreased the activity of superoxide dismutase and catalase, and lowered free proline levels and H_2_O_2_ concentrations, compared to the nontreated plants [4].

Grašič et al. [88] reported that the combination of UV and drought affects Si concentrations in leaves. Proso millet (*Panicum miliaceum* L.) under ambient UV radiation and water shortage had significantly lower Si levels in leaves, compared to plants under reduced UV radiation, which was connected to lowered transpiration rate.

As summarized on Table 3, Si addition to UV-B and drought stress reduces membrane damage, oxidative stress and levels of some antioxidants or antioxidant enzymes activities.

## 6. Conclusions

Silicon is an abundant chemical element that provides limited but important contributions to the normal functionality of plants during stress. Deciphering the mechanisms of how silicon contributes to abiotic stress alleviation is still a great challenge to plant biologists. Based on the literature included here, Si can either increase or decrease the contents of different molecules or parameters in plants under drought and/or UV-B stress conditions. Studies have shown that Si reduces the harmful effects of UV radiation through increased antioxidant capacity, anthocyanin and soluble sugars levels, the uptake of some nutrients, total biomass of plants, cell lignification and suberinization. Si also decreases the content of phenol substances and osmolyte leakage. In relation to drought, Si increases plant growth, the number of leaves, turf quality, root:shoot ratio, expression of photosynthesis-related genes, relative water content, leaf water content, the contents of some plant hormones, the activity of ascorbate peroxidase, and the levels of ascorbic acid, glutathione and carotenoids.

Si treated plants respond similarly to drought or UV-B stress. Addition of Si in plants exposed to increased UV-B radiation and drought increases photosynthesis rate and chlorophyll content, decreases the activity of catalase and superoxide dismutase, and decreases the content of malondialdehyde. Therefore, plants contain less reactive oxygen species.

Knowledge of the combined effects of drought and UV-B radiation is fundamental here, because these usually occur together. The combination of those two stress factors has not been studied in detail yet, but the first studies have showed some mitigating effects of Si.

## Figures and Tables

**Table 3:** Effects of silicon supplementation on the measured parameters in the presence of UV-B, drought and their combination as revised from the literature. Arrows indicate increases (↑) or decreases (↓) of each parameter under UV-B, drought, or combined stress conditions (UV-B × drought) in silicon treated plants.

			Drought	UV-B	UV-B × Drought	Reference
Elemental composition		Nutrient uptake	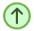			[43]
		P, Mg uptake		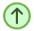		[86]
		Leaf Si concentration		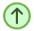		[3]
		Leaf C:N ratio	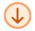			[49]
Antioxidant capacity				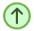		[3]
Antioxidants	Non-enzymatic system	Flavonoids		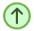 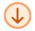		[3](↑), [5](↓)
		Anthocyanin		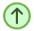		[3]
		Ascorbic acid	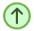			[46]
		Glutathione	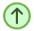			[46]
		Proline	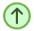 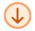	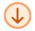	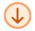	[44,46,47] ^1^[4][4]
		Phenol substances		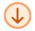		[5]
		Carotenoids	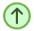			[6,48]
		Soluble sugars		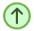		[3]
	Enzymatic system	Catalase	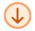	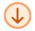	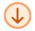	[6][85][4]
		Superoxide dismutase	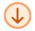	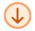	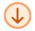	[6][85][4]
		Peroxidase	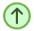	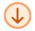		[47][85]
		Ascorbate peroxidase	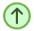			[44]
Membrane damage		Malondialdehyde	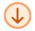	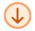	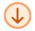	[44,46][3,4][4]
		Osmolyte leakage		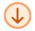	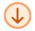	[4][4]
Hormones		Indole-3-acetic acid	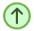			[6]
		Gibberellic acid	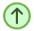			[6]
		Cytokinins	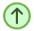			[6]
		Abscisic acid	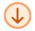			[6]
Oxidative stress		Reactive oxygen species	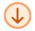	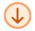		[45][5]
		H_2_O_2_			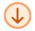	[4]
Photosynthesis		Photosynthesis rate	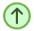	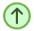		[45,46,49,50,51][4]
		Chlorophyll content	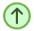	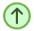		[6,48][3]
		Photosynthesis-related genes expression	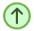			[48]
Morphology		Growth rate	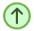			[6,46,48,49,52]
		Number of leaves	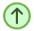			[46,52]
		Total biomass		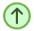		[3,84]
		Root:shoot ratio	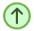			[49]
		Turf quality	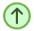			[49]
Water content		Relative water content	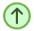			[6,46]
		Leaf water content	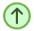			[49]
Cell structure		Lignification, suberinisation		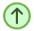		[84]

^1^ [46](↑), [47](↓).

## Data Availability

Not applicable.
